# A case of superior mesenteric artery stenting for small intestinal ischaemia caused by superior mesenteric artery invasion of pancreatic cancer

**DOI:** 10.1093/bjrcr/uaae031

**Published:** 2024-08-26

**Authors:** Hiroshi Kuwamura, Yohsuke Suyama, Yasuhiro Enjoji, Takahiro Einama, Yoji Kishi, Hiroshi Shinmoto

**Affiliations:** Department of Radiology, National Defense Medical College, Saitama 359-8513, Japan; Department of Radiology, National Defense Medical College, Saitama 359-8513, Japan; Department of Radiology, National Defense Medical College, Saitama 359-8513, Japan; Department of Hepato-Biliary-Pancreatic Surgery, National Defense Medical College, Saitama 359-8513, Japan; Department of Hepato-Biliary-Pancreatic Surgery, National Defense Medical College, Saitama 359-8513, Japan; Department of Radiology, National Defense Medical College, Saitama 359-8513, Japan

**Keywords:** superior mesenteric artery stent, intestinal ischaemia, pancreatic cancer, re-stenosis, tumour ingrowth, neoplastic vascular obstruction

## Abstract

Superior mesenteric artery (SMA) invasion by a malignant tumour is a serious condition leading to intestinal ischaemia. Although SMA stenting has been reported to be useful for SMA dissection and stenosis caused by atherosclerotic plaque, SMA stenting for stenosis caused by malignant tumour invasion is rarely reported and uncertain. A 75-year-old woman presented intestinal ulcer and melena caused by SMA invasion of unresectable pancreatic cancer. The bare metal stent was implanted for the vessel stenosis, and a small intestinal ulcer was markedly improved after stenting. However, one and a half months after stenting the stent was occluded and a thrombectomy was performed. After thrombectomy, residual stenosis caused by tumour invasion was observed in the stent. The patient suddenly died 2 days after thrombectomy before additional covered stenting for residual stenosis. Stent implantation may be a treatment option for intestinal ischaemia caused by vessel invasion of malignant tumours. On the other hand, re-stenosis of the stent due to tumour ingrowth is a problem, and covered stenting is considered for long-term stent patency.

## Background

Superior mesenteric artery (SMA) invasion by a malignant tumour is a serious condition leading to intestinal ischaemia. Although SMA stenting has been reported to be useful in SMA dissection and stenosis caused by atherosclerotic plaque,^1^^,^^2^ SMA stenting for stenosis caused by malignant tumour invasion is rarely reported and uncertain. This report describes a case in which SMA stenting was performed to treat intestinal ischaemia due to SMA invasion of pancreatic cancer.

## Clinical presentation

**Figure 1.**
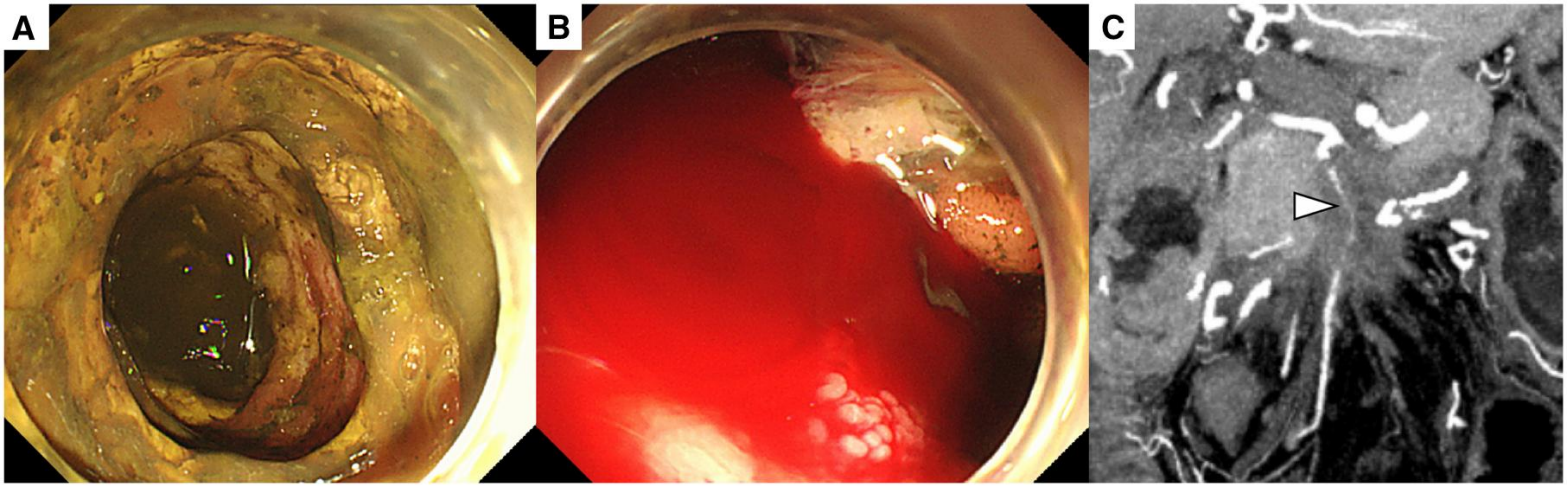
(A, B) Endoscopic images before superior mesenteric artery (SMA) stent placement show multiple ulcers, mucosal erosions and intestinal haemorrhage due to remaining small bowel ischaemia. (C) Coronal contrast-enhanced computed tomography (CECT) image before SMA stenting shows a highly narrowed SMA (arrowhead) due to invasion of pancreatic cancer.

A 75-year-old woman presented with unresectable pancreatic cancer. The patient underwent partial resection of the small intestine because of necrosis caused by SMA invasion of the tumour. SMA invasion progressed and caused further ischaemia in the remaining small intestine, resulting in a refractory small intestinal ulcer and haemorrhage (Figure 1A and B), and a low haemoglobin level of 4.8 g/dL. Contrast-enhanced computed tomography (CECT) revealed pancreatic cancer surrounding the SMA, which was highly narrowed (Figure 1C). Because further small bowel resection may result in short bowel syndrome, SMA stenting was planned to improve the remaining small bowel ischaemia.

## SMA stenting procedure

**Figure 2.**
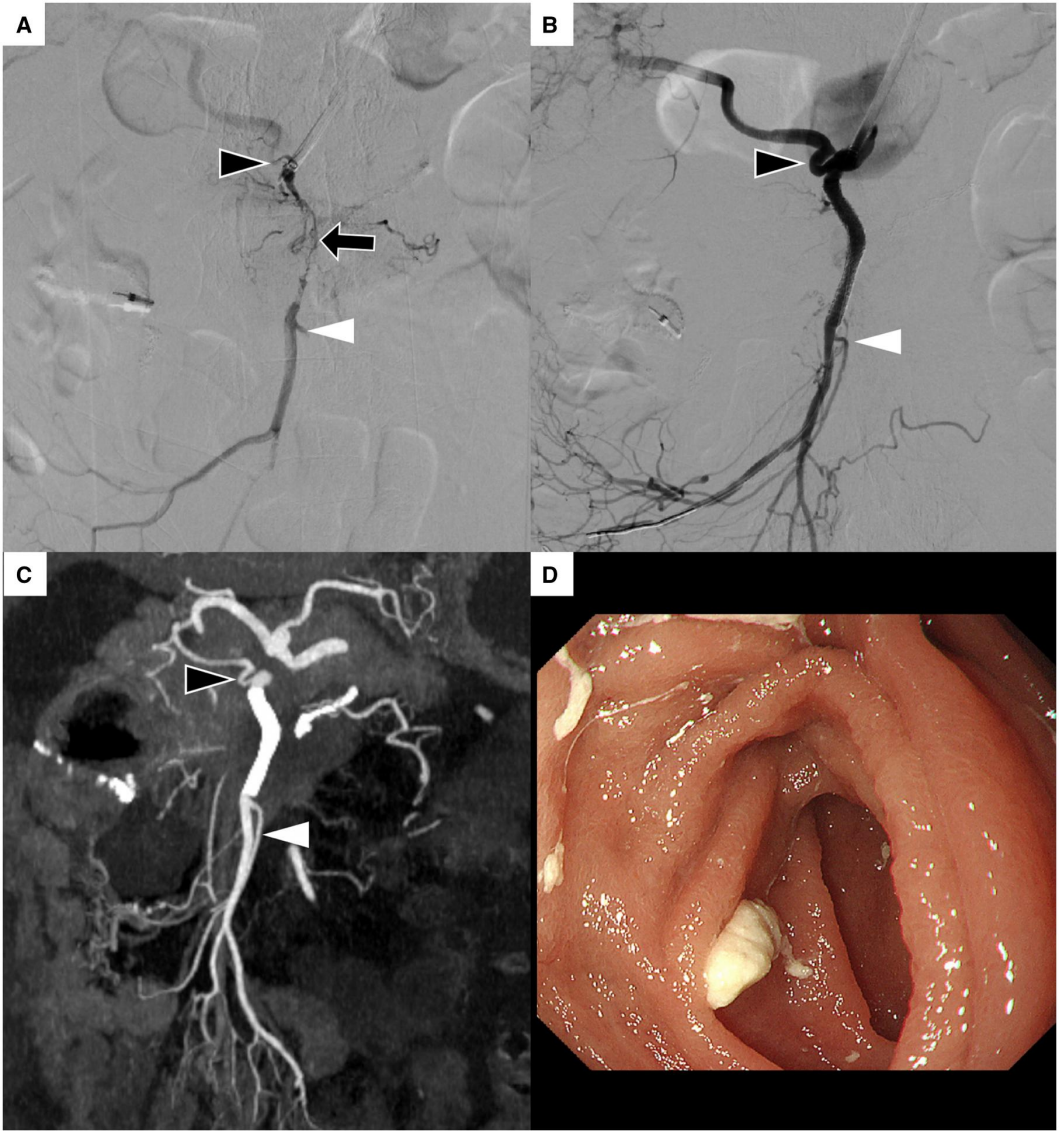
(A) Superior mesenteric artery (SMA) angiography shows severe and long stenosis (black arrow) at the area of tumour invasion. Right hepatic artery (black arrowhead) and ileal artery (white arrowhead) originate near stenosis. (B, C) SMA angiography and coronal contrast-enhanced computed tomography (CECT) images after stenting show stent patency and improvement of intestinal blood flow. Right hepatic artery (black arrowhead) and ileal artery (white arrowhead) are patent without stent covering. (D) Endoscopic image after SMA stent placement shows the disappearance of multiple ulcers, mucosal erosions and intestinal haemorrhage caused by bowel ischaemia.

The left brachial artery was punctured under ultrasound guidance, and a 6 Fr guiding sheath (6 Fr 90 cm Destination, Terumo, Tokyo, Japan) was placed in the abdominal aorta. After placement of the sheath, 2000 units of heparin were injected intravenously. 5 Fr catheter (HHT, Medikit, Tokyo, Japan) and 0.035″ guidewire (radifocus, Terumo, Tokyo, Japan) were used for cannulation of SMA. SMA angiography revealed severe and long stenosis at the area of the tumour invasion, right hepatic artery and ileal artery were branched near the stenosis (Figure 2A). A 0.014″ guidewire (ASAHI CHIKAI V, Asahi Intec, Tokyo, Japan) was used to pass the stenosis and three bare stents (4 mm × 19 mm, 5 mm × 19 mm, and 6 mm × 15 mm Express Vascular SD, Boston Scientific, MN, United States) were implanted, overlapping from distal to proximal of the lesion without covering the origin of important side branches. Angiography of the SMA after stenting showed good patency (Figure 2B).

## Outcome of SMA stenting

The patient underwent double antiplatelet therapy combining aspirin 100 mg/day and clopidogrel 75 mg/day after stenting. Coronal CECT 1 week after stenting confirmed stent patency (Figure 2C). Endoscopy performed 10 days after stenting showed marked improvement of the small intestinal ulcer and mucosal erosions (Figure 2D). Haemoglobin level improved from 4.8 to 9.9 g/dL, and the patient was discharged 13 days after stenting. The stent remained patent, and no symptoms of intestinal ischaemia were observed for more than 1 month after discharge. However, one and a half months after stenting, the patient developed sudden abdominal pain, and CECT showed occlusion of the SMA stent. Therefore, a thrombectomy was performed.

## SMA thrombectomy procedure

**Figure 3.**
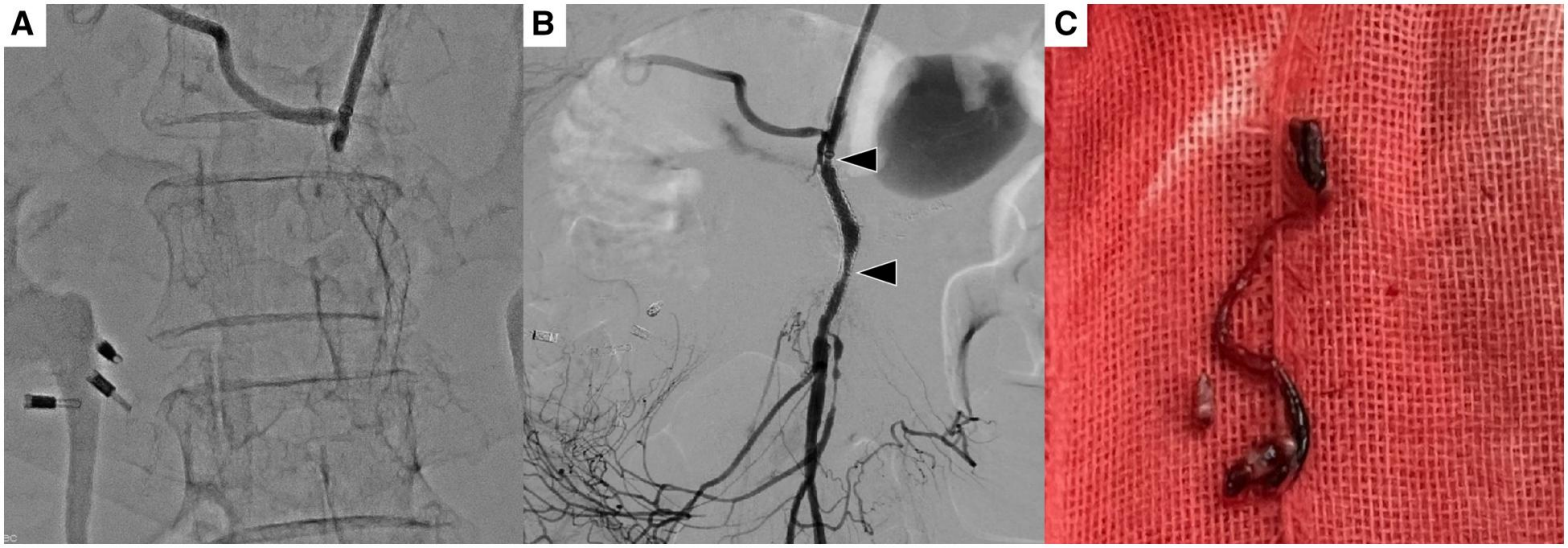
(A) Superior mesenteric artery (SMA) angiography shows complete occlusion of the stent. (B) Thrombectomy is performed and SMA angiography shows that the stent is patent; however, there is residual stenosis in the middle and proximal of the stent (black arrowheads). (C) This is a thrombus retrieved by SMA thrombectomy.

A 6 Fr guiding sheath (Destination; Terumo, Tokyo, Japan) was inserted into the SMA from the left brachial artery and SMA angiography revealed complete occlusion of the stent (Figure 3A). A 6 Fr large lumen guiding catheter (Cerulean DD6; Medikit, Tokyo, Japan) was used for the thrombectomy. The clot was retrieved and abdominal pain immediately improved. SMA angiography after thrombectomy showed recanalization of the stent and residual stenosis in the middle and proximal portions of the stent (Figure 3B).

## Final outcome of this case

Additional covered stent for re-stenosis was planned as a standby procedure because optimal size covered stent was not immediately available in our hospital; however, the patient suddenly went into cardiopulmonary arrest 2 days after thrombectomy and died before additional covered stenting.

## Discussion

SMA stenting has been reported to be effective in SMA dissection and atherosclerotic stenosis. Fu et al^1^ reported that nine patients with symptomatic isolated SMA dissection were treated with bare metal stents, and the false lumen was stable or shrunken in all cases. Girault et al^2^ placed covered stents in 86 patients with SMA stenosis and reported a high technical success rate of 97% with a primary patency rate of 83% and a secondary patency rate of 99% at 1 year. Covered stenting has also been reported to be useful in the treatment of ruptured SMA pseudoaneurysms, allowing haemostasis while preserving the intestinal blood flow.^3^ This article reports the case of small bowel ischaemia caused by malignant tumour invasion of the SMA that was treated with SMA stenting; however, similar case reports of SMA stenting for malignant vessel obstruction are rarely reported and the effectiveness is uncertain.

In a multivariate analysis of the risk factors for SMA stent edge stenosis, the diameter ratio of the stent to the vessel and the angle of the distal edge were reported as risk factors for stent edge stenosis, suggesting that the implantation of a stent that is compatible with the vessel diameter is important for long-term stent patency.^4^^,^^5^ In our case, stent placement was performed by overlapping the three bare metal stents (4 mm at the distal end of the stenosis, 5 mm in the middle, and 6 mm at the proximal end of the stenosis), which conformed the vessel calibre between distal 4 mm and proximal 6 mm vessel diameter. Although a bare metal stent with a larger diameter may be desirable to prevent early stent occlusion, placement of an over-sized balloon-expandable bare metal stent larger than the distal 4 mm vessel diameter could cause vascular injury. Therefore, a 4 mm balloon-expandable bare metal stent of the same size as the distal vessel diameter was placed. The covered stent was not used for the following reasons: (1) there was a risk of occluding the right hepatic artery and side branches of ileal artery originating near the stenosis which is very important for hepatic and intestinal blood flow, (2) covered stent implantation through severe and long malignant stenosis was expected to be technically difficult because of high-profile delivery shaft compared with the low-profile delivery shaft of the bare metal stent, (3) covered stent optimised for vessel diameter and lesion length was not immediately available in our hospital. Therefore, balloon-expandable bare metal stents were placed, which can easily and accurately adjust the stent position, accommodate a variety of vessel diameters, have a lower risk of branch occlusion, and can easily deliver through severe malignant stenosis.

Neointimal thickening within the stent is a common cause of stent re-stenosis^6^; however, tumour ingrowth into the stent should also be considered as a cause of stent re-stenosis in the case of vessel obstruction due to malignant tumour.^7^^,^^8^ The covered stent is effective for neoplastic vascular occlusion because tumour ingrowth does not occur, whereas there is a risk of occluding important side branches. In our case, acute stent occlusion may have occurred because of thromboembolism triggered by tumour invasion into the stents. Additional covered stents which can prevent tumour invasion should be placed for residual stenosis in the stent after thrombectomy; however, an appropriate covered stent adapted to vessel diameter and lesion length without occluding side branches could not be prepared immediately in our hospital.

We experienced a case of refractory intestinal ischaemia and haemorrhage caused by SMA invasion of malignant tumour treated with SMA bare metal stent implantation, and intestinal ulcers due to intestinal ischaemia improved. On the other hand, re-stenosis and occlusion of the stent due to malignant tumour invasion is a problem, and covered stenting is considered for long-term stent patency.

## Learning points

- SMA stenting is a treatment option for bowel ischaemia caused by malignant vessel invasion.
- Covered stent is considered to avoid re-stenosis and occlusion caused by tumour ingrowth.
